# The Vitamin D Status in Inflammatory Bowel Disease

**DOI:** 10.1371/journal.pone.0101583

**Published:** 2014-07-03

**Authors:** Lauren Elizabeth Veit, Louise Maranda, Jay Fong, Benjamin Udoka Nwosu

**Affiliations:** 1 Department of Pediatrics, University of Massachusetts Medical School, Worcester, Massachusetts, United States of America; 2 Department of Quantitative Health Sciences, University of Massachusetts Medical School, Worcester, Massachusetts, United States of America; University of Tennessee, United States of America

## Abstract

**Context:**

There is no consensus on the vitamin D status of children and adolescents with inflammatory bowel disease (IBD).

**Aim:**

To determine the vitamin D status of patients with IBD by comparing their serum 25(OH)D concentration to that of healthy controls.

**Hypothesis:**

Serum 25(OH)D concentration will be lower in patients with IBD compared to controls.

**Subjects and Methods:**

A case-controlled retrospective study of subjects with IBD (n = 58) of 2–20 years (male n = 31, age 16.38±2.21 years; female n = 27, age16.56±2.08 years) and healthy controls (n = 116; male n = 49, age 13.90±4.59 years; female n = 67, age 15.04±4.12years). Study subject inclusion criteria: diagnosis of Crohn’s disease (CD) or ulcerative colitis (UC). Vitamin D deficiency was defined as 25(OH)D of (<20 ng/mL) (<50 nmol/L), overweight as BMI of ≥85^th^ but <95^th^ percentile, and obesity as BMI ≥95^th^ percentile. Data were expressed as mean ± SD.

**Results:**

Patients with CD, UC, and their controls had mean serum 25(OH)D concentrations of 61.69±24.43 nmol/L, 53.26±25.51, and 65.32±27.97 respectively (ANOVA, *p* = 0.196). The overweight/obese controls had significantly lower 25(OH)D concentration compared to the normal-weight controls (p = 0.031); whereas 25(OH)D concentration was similar between the normal-weight and overweight/obese IBD patients (p = 0.883). There was no difference in 25(OH)D between patients with UC and CD, or between subjects with active IBD and controls. However, IBD subjects with elevated ESR had significantly lower 25(OH)D than IBD subjects with normal ESR (p = 0.025), as well as controls (65.3±28.0 nmol/L vs. 49.5±25.23, p = 0.045).

**Conclusion:**

There is no difference in mean serum 25(OH)D concentration between children and adolescents with IBD and controls. However, IBD subjects with elevated ESR have significantly lower 25(OH)D than controls. Therefore, IBD subjects with elevated ESR should be monitored for vitamin D deficiency.

## Introduction

There is no consensus on the vitamin D status of children and adolescents with inflammatory bowel disease (IBD).The composite term IBD refers to two diseases, Crohn’s disease (CD) and ulcerative colitis (UC), which are characterized by chronic inflammation of the gastrointestinal tract, marked by recurrent periods of remission and exacerbation [1]. Pediatric CD is characterized by discontinuous, transmural inflammation of the gastrointestinal tract with preferential involvement of the ileo-colonic segment, while UC is characterized by a more superficial, continuous inflammation that extends proximally from the rectum to variable areas of the large intestine [1].

Prolonged vitamin D deficiency could lead to poor health, as strong associations between vitamin D deficiency and increased risk for several diseases such as type 1 and type 2 diabetes, cardiovascular diseases, rheumatoid arthritis, infectious diseases, depression, and cancers of the breast, prostate, colon, and pancreas, have been reported [2]–[4]. Though there is an ongoing debate on the significance of these extra-skeletal functions of vitamin D in humans [5], [6], there is a universal consensus on its skeletal functions, as vitamin D has been demonstrated to be vital for bone mineralization, maintenance of bone strength, and the prevention of fractures and consequent immobilization [6], [7].

The lack of consensus on the vitamin D status of children and adolescents with IBD stems from two primary reasons: first, the studies that investigated the vitamin D status of children and adolescents with IBD [8]–[12] focused primarily on determining the prevalence of vitamin D deficiency in IBD, and secondarily on comparing the vitamin D status of patients with the subtypes of IBD, i.e., UC and CD, but failed to compare their results directly to a local control group of healthy children and adolescents.

Secondly, only one study has directly compared the vitamin D status of children and adolescents with IBD to the vitamin D status of age- and gender-matched peers [13]. This cross-sectional study of 60 children with newly-diagnosed IBD and 56 controls found that 25(OH)D level was significantly lower in children with IBD compared to the controls. However, no subsequent studies have been performed to confirm these findings. Furthermore, to our knowledge, there has been no case-controlled study examining the vitamin D status of patients with established IBD of more than one year duration. This lack of clarity on the vitamin D status of children and adolescents with IBD compared to their peers has made it difficult to propose a coherent recommendation for vitamin D supplementation in patients with IBD [9], [14].

We designed this study to explore the hypothesis that serum 25(OH)D concentration is significantly lower in patients with IBD compared to controls. The primary aim of this study was to characterize the vitamin D status of patients with IBD by directly comparing their mean serum 25(OH)D concentration to that of a local group of healthy children.

## Materials and Methods

### Ethics statement

The study protocol was approved by the University of Massachusetts Institutional Review Board. All patient records and information were anonymized and de-identified prior to analysis.

### Subjects

All data were sourced from the Children’s Medical Center Database of the UMassMemorial Medical Center, Worcester, Massachusetts, USA. The medical records of children and adolescents of ages 2–20 years with a confirmed diagnosis of Crohn’s disease or ulcerative colitis from January 1, 2007 through June 30, 2013, were reviewed. Study subjects (n = 58; 31 males) were included if they had a diagnosis of Crohn’s disease or ulcerative colitis. Subjects’ height, weight, gender, race, IBD diagnosis, date of endoscopic IBD diagnosis, and any history of vitamin D supplementation were recorded.

A group of healthy peers served as controls. The controls were identified from the same database as the subjects. Subjects were included in the control group (n = 116; 49 males) if they carried no diagnosis of Crohn’s disease or ulcerative colitis. Subjects’ height, weight, gender, race, and history of vitamin D supplementation were similarly recorded.

Patients were excluded from this study if they carried a concurrent diagnosis of any disease that affects calcium or vitamin D metabolism. Subjects with a malabsorption syndrome, other than IBD, were excluded. Further exclusion criteria included patients with a history of vitamin D or calcium supplementation prior to the date of 25(OH)D measurement, subjects on continuous doses of oral corticosteroids for the management of any disease other than IBD, pregnant or lactating subjects, and patients with chronic liver disease.

We identified 76 children and adolescents of ages 2–20 years with a diagnosis of IBD. Eighteen subjects were excluded based on the above exclusion criteria. Fifty-eight subjects were included in the study. The control group consisted of 116 non-IBD peers who were randomly drawn from the same database using a systematic sampling scheme. For this method, we alphabetized the list of control patients then selected every 5^th^ patient for inclusion in our control group, thereby preserving randomization.

The ages of both the study subjects and their controls were determined by the date of 25(OH)D measurement. The duration of disease was designated as the interval from the date of endoscopic diagnosis of IBD to the date of 25(OH)D measurement. The percentages of subjects from the various ethnic/racial groups for the control group were as follows: Non-Hispanic 75%, African American 8%, White Hispanic 5%, Multi-ethnicity 5%, Unknown 4%. Similarly, the percentages from the various ethnic/racial groups for the IBD groups were: Non-Hispanic white 85%, African American 3%, White Hispanic 3%, Multi-ethnicity 3%, Unknown 5%.

Because vitamin D status varies with sunlight exposure and the seasons, we categorized each subject’s date of vitamin D draw according to the seasons as follows: fall (September 22–December 21), winter (December 22–March 21), spring (March 22–June 21), and summer (June 22–September 21)[15].

### Anthropometry

Height was measured to the nearest 0.1 cm using a wall-mounted stadiometer (Holtain Ltd, Crymych, Dyfed, UK) that was calibrated daily. Weight was measured to the nearest 0.1 kg using an upright scale. BMI was derived using the formula weight/height^2^ (kg/m^2^), and expressed as standard deviation score (SDS) for age and gender based on National Center for Health Statistics (NCHS) data [16]. Overweight was defined as BMI of ≥85^th^ but <95^th^ percentile, while obesity was defined as a BMI of ≥95^th^ percentile for age and gender.

### Assay

Serum 25(OH)D concentration was analyzed using 25-hydroxy chemiluminescent immunoassay (DiaSorin Liaison; Stillwater, Minnesota), which has a 100% cross-reactivity with both metabolites of 25(OH)D namely, 25(OH)D_2_ and 25(OH)D_3_ and thus measures total serum 25(OH)D content. Its functional sensitivity is 10 nmol/L, and its intra- and inter-assay coefficients of variation are 5% and 8.2%, respectively. Vitamin D status was defined using 25(OH)D values based on criteria by The Endocrine Society Clinical Practice Guideline as follows: vitamin D deficiency<20 ng/mL (50 nmol/L), insufficiency 20–29.9 ng/mL (50–74.5 nmol/L), and sufficiency≥30 ng/mL (75 nmol/L)[5], which is similar to the classification of vitamin D status by the American Academy of Pediatrics and the Institutes of Medicine criteria which denote vitamin D deficiency as 25(OH)D <50 nmol/L; and sufficiency as 25(OH)D >50 nmol/L [6], [17].

### Statistical analyses

Statistical analyses were performed using the SPSS Predictive Analytics SoftWare v.21 (IBM Corporation, Armonk, NY) and Microsoft Excel (2007). Means and standard deviations were calculated for descriptive summary statistics and 25(OH)D measurements. Multivariate and univariate comparisons on anthropometrics, 25(OH)D, and other variables were conducted using ANOVA and two-tailed student’s t-test respectively. Specifically, ANOVA was used to compare the differences in the parameters of interests between the controls, UC, and CD subjects. Height, weight, and BMI data were expressed as z-scores. Race, gender proportionality, and seasons of blood draw were compared using Fisher’s exact test. Data were expressed as mean ± standard deviation (SD).

## Results

### Comparative analysis of the characteristics of subjects with UC, CD, and their controls

Table 1 shows the analysis of the characteristics of the subjects with UC, CD, and controls using a one-way ANOVA. The controls were younger, had higher value for weight SDS, and a higher prevalence of overweight/obese status compared to the UC and CD groups. There was no difference in mean serum 25(OH)D concentration (p = 0.196) between the groups. There was a non-significantly higher prevalence of vitamin D deficiency as defined by a 25(OH)D value of <50 nmol/L (20 ng/mL) in both the UC and CD compared to controls (p = 0.070).

**Table 1:** A Comparative Analysis of the Characteristics of Subjects with Ulcerative Colitis, Crohn’s Disease, and Healthy Controls.

Parameter	CD	95% CI	UC	95% CI	Controls	95% CI	*p*
Total	40	-	18	-	116	-	-
Age (years)	16.61±2.20	(15.91, 17.32)	16.13±1.99	(15.14, 17.12)	14.56±4.35	(13.76, 15.36)	0.008
Height z-score	−0.63±1.18	(−1.01, −0.25)	0.00±0.98	(−0.49, 0.49)	−0.02±1.42	(−0.28, 0.24)	0.040
Weight z-score	−0.31±1.45	(−0.77, 0.16)	0.30±1.11	(−0.26, 0.85)	0.40±1.60	(0.11, 0.70)	0.041
BMI z-score	−0.08±1.35	(−0.51, 0.35)	0.28±0.99	(−0.21, 0.78)	0.48±1.38	(0.23, 0.74)	0.076
Sex (% males)	24/40 (60.0%)	-	7/18 (38.9%)	-	49/116 (42.2%)	-	0.124
Race (% white)	36/40 (90.0%)	-	15/18 (83.3%)	-	87/116 (75.0%)	-	0.118
Season (%Winter-Spring)	17/40 (42.5%)	-	8/18 (44.4%)	-	51/116 (44.0%)	-	0.985
BMI status (% overweight/obese)	11/40 (27.5%)	-	2/18 (11.1%)	-	45/116 (38.8%)	-	0.046
Disease duration (years)	2.61±2.76	(1.68, 3.54)	2.76±2.54	(1.49, 4.02)	-	-	0.854
Mean serum 25(OH)D (nmol/L)	61.69±24.43	(53.88, 69.50)	53.26±25.51	(40.57, 65.94)	65.32±27.97	(60.18, 70.46)	0.196
25(OH)D ≤15 ng/mL (%)	6/40 (15.0%)	-	5/18 (27.8%)	-	12/116 (10.3%)	-	0.118
25(OH)D ≤20 ng/mL (%)	16/40 (40.0%)	-	9/18 (50.0%)	-	31/116 (26.7%)	-	0.070
25(OH)D ≤30 ng/mL (%)	29/40 (72.5%)	-	15/18 (83.3%)	-	87/116 (75%)	-	0.671

SDS standard deviation score; 25(OH)D 25-hydroxyvitamin D.

### Comparison of the characteristics of the subjects with UC vs. CD

When the IBD cohort was stratified by IBD sub-types, UC vs. CD, there were no significant differences in age, gender, weight, BMI, disease duration, or season of vitamin D measurement. Subjects with CD were non-significantly shorter than the UC patients (p = 0.05). There was no difference in mean serum 25(OH)D concentration between groups (53.3±25.4 vs. 61.8±24.4, p = 0.24). Subjects with UC had a non-significantly higher prevalence of vitamin D deficiency compared to the CD subjects (50% vs. 40%, p = 0.53).

### The effect of adiposity on serum 25(OH)D in IBD vs. controls

**Figure 1:**
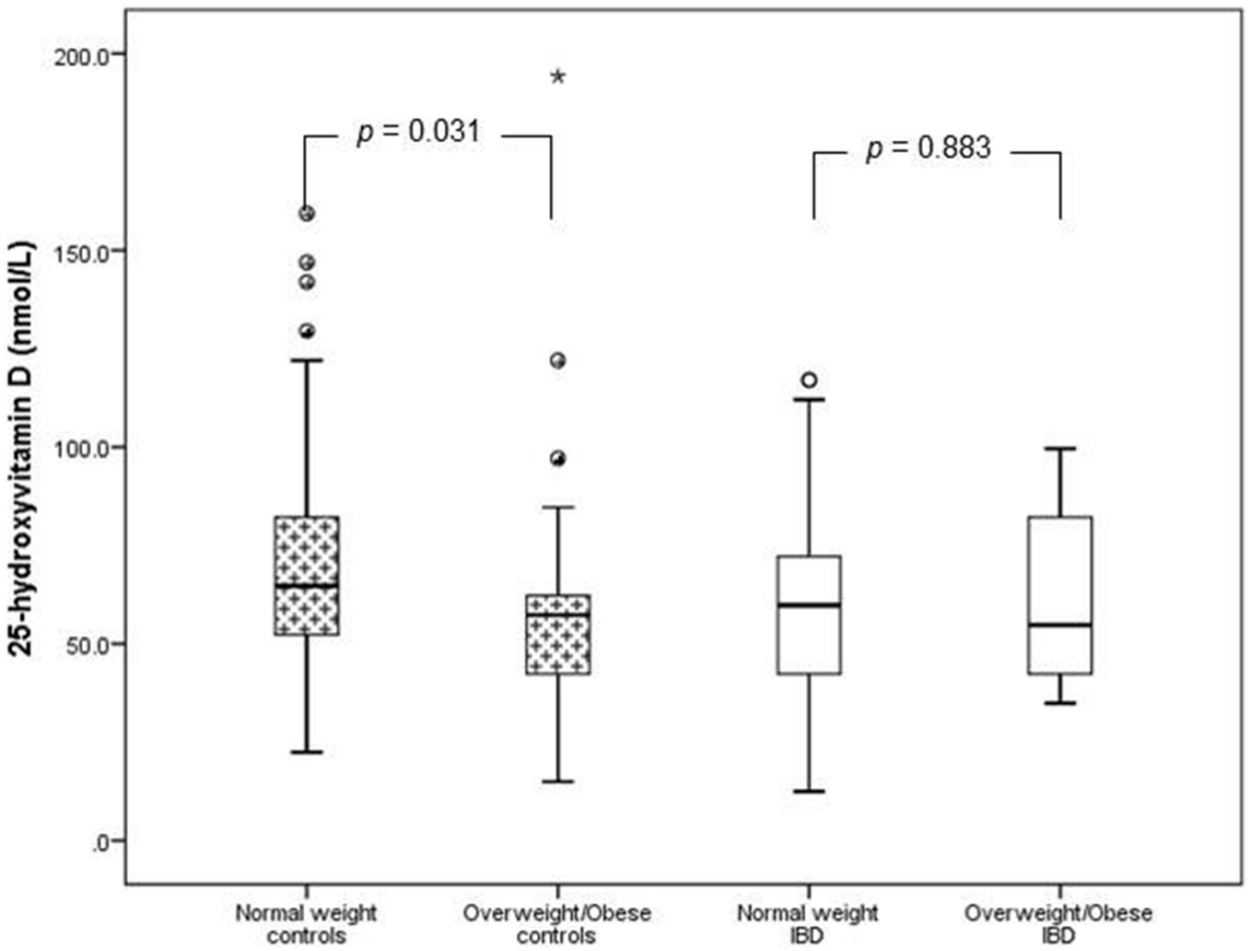
Box plots of the comparison of 25-hydroxyvitamin D concentration of patients with inflammatory bowel disease (IBD) and normal controls stratified by body mass index. This figure shows that the overweight/obese controls had significantly lower value for 25(OH)D than the normal weight controls (58.32±27.63 vs. 69.76±27.45, p = 0.031), while there was no significant difference in 25(OH)D value between the normal weight and overweight/obese IBD patients (59.71±26.44 vs. 60.91±23.26, p = 0.883). Note: 50 nmol/L = 20 ng/mL.

To investigate the effect of adiposity on 25(OH)D concentration in IBD vs. controls, the subjects were stratified into normal-weight vs. overweight/obese groups (Figure 1). The overweight/obese controls had significantly lower 25(OH)D concentration compared to the normal-weight controls (p = 0.031), whereas 25(OH)D concentration was similar between the normal-weight and overweight/obese IBD patients (p = 0.883). Further stratification of the IBD cohort into UC and CD showed no difference in 25(OH)D concentration between the normal-weight and overweight/obese groups for CD (p = 0.98) or UC (p = 0.70). These data suggest that adiposity has no effect on serum 25(OH)D concentration of patients with IBD.

### The relationship between the duration of IBD and serum 25(OH)D concentration

**Figure 2:**
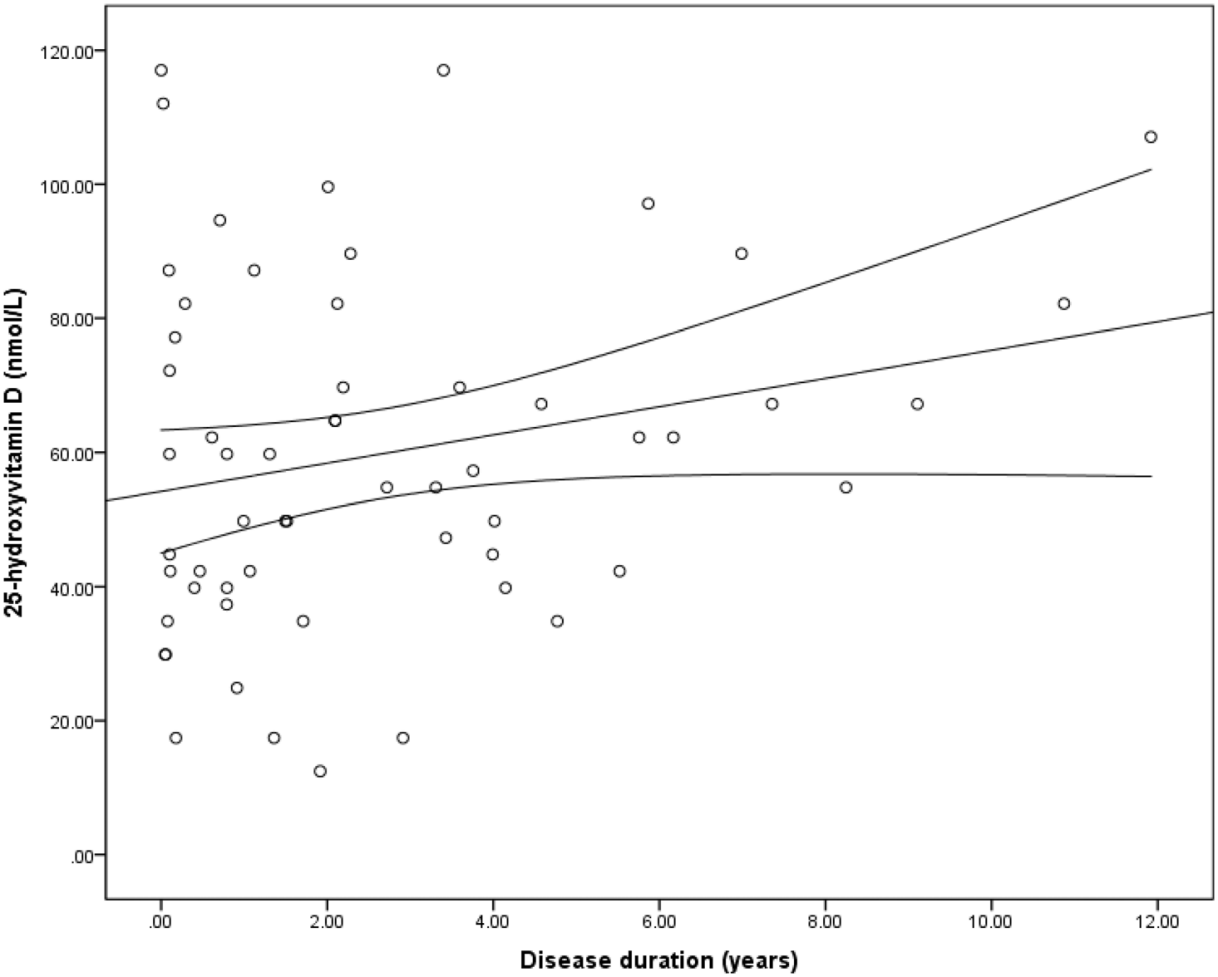
Scatterplot of the comparison of the 25-hydroxyvitamin D concentration and the duration of disease in inflammatory bowel disease. This figure shows a non-significant positive relationship between serum 25(OH)D concentration and the duration of disease (r^2^ = 0.054, β = 0.23, p = 0.08).

We first compared the mean 25(OH)D concentration of the control group to 13 patients with IBD who had serum 25(OH)D estimation at the time of diagnosis of IBD, i.e. during active disease, and found no significant difference in their mean 25(OH)D concentration (65.3±28.0 vs. 63.8±30.1 nmol/L, p = 0.86). Next, we investigated the relationship between the duration of disease and serum concentration of 25(OH)D (Figure 2). There was a non-significant, positive relationship between serum 25(OH)D concentration and the duration of disease (r^2^ = 0.054, β = 0.23, p = 0.08).

### The relationship between the severity of IBD and serum 25(OH)D concentration

Using the Pediatric Crohn’s Disease Activity Index (PCDAI)[18] and Lichtiger Colitis Activity Index (LCAI)[19], to quantify the severity of IBD, we investigated the differences in serum 25(OH)D concentration in the three groups: controls, n = 116; quiescent IBD cases n = 22; active IBD cases n = 8. There was no difference in serum 25(OH)D concentration between three groups: [controls, 65.3±27.7 nmol/L; quiescent IBD cases 61.3±28.3; active IBD cases 54.5±22.5, ANOVA p  = 0.498. Post hoc comparisons detected no significant difference in 25(OH)D levels between the groups.

We then stratified the IBD cohort using ESR as a marker of inflammation and compared their serum 25(OH)D level to the controls. Within the IBD cohort, serum 25(OH)D concentration was significantly lower in patients with elevated ESR levels (ESR of >21 mm/hr) compared to those with normal ESR values (49.5±25.2 nmol/L vs. 65.6±22.1, p = 0.025).

Subsequent analysis of the mean serum 25(OH)D concentrations of the 3 groups: controls (n = 116), IBD with elevated ESR (n = 19), and IBD with normal ESR (n = 37), showed a near-significant difference in 25(OH)D between the groups (ANOVA p = 0.052). Post hoc comparisons showed a significant difference in 25(OH)D concentration between the controls and IBD subjects with elevated ESR (65.3±28.0 nmol/L vs. 49.54±25.23, p = 0.045), but not between the controls and the IBD subjects with normal ESR (65.3±28.0 vs. 65.6±22.1, p = 0.998).

## Discussion

This study found no significant difference in the serum concentration of 25(OH)D in children and adolescents with IBD compared to normal controls. The normal-weight controls had significantly higher 25(OH)D concentration compared to the overweight/obese controls as well as the normal-weight IBD subjects. There was no difference in 25(OH)D between the normal-weight IBD and the overweight/obese IBD subjects. In contrast, subjects with IBD and elevated ESR had significantly lower serum 25(OH)D concentration compared to the healthy controls, and subjects with IBD and normal ESR level.

This is the second study to directly compare the vitamin D status of children and adolescents with IBD to healthy controls. Our finding, however, is contrary to the report by El-Matary et al [13] who described lower 25(OH)D concentration in a cross-sectional study of children with newly-diagnosed IBD compared to controls. Our report differed from the above-referenced study in that our study included subjects with more established IBD, with mean disease duration of thirty-two months. However, our sub-analysis found no difference in the mean 25(OH)D concentration measure at the time of IBD diagnosis compared to the mean 25(OH)D of the controls. Thus, this study found no evidence for subnormal vitamin D status in patients with newly-diagnosed IBD compared to controls in our cohort.

This study’s findings on the 25(OH)D levels of established IBD are consistent with the report of a case-controlled study that reported no significant difference in serum 25(OH)D in adult patients with established IBD [20].

The effect of disease duration on vitamin D deficiency is unclear, as some studies report longer disease duration as a risk factor for vitamin D deficiency [20], while others found a positive correlation between disease duration and serum 25(OH)D concentration [8]. This study’s findings are in agreement with the above report of a positive relationship between disease duration in IBD and 25(OH)D concentration (Figure 2). One explanation for this association is that the initiation of treatment in patients with IBD results in some degree of healing of the mucosal damage and consequent improvement in the absorption of vitamin D. It is also possible that the phenomenon of compensation which has been described in celiac disease, also occurs in IBD and leads to increased vitamin D absorptive capacity by the unaffected mucosal surfaces [21].

Additional case-controlled studies are warranted to accurately characterize the vitamin D status of patients with IBD at various phases of the disease. This is necessary because the other studies that have examined the vitamin D status of patients with IBD lacked control groups, focused primarily on either the prevalence rate of vitamin D deficiency in IBD, or limited their comparison of vitamin D status to patients with the subtypes of IBD, i.e., UC and CD. For example, one study found normal 25(OH)D concentration in children with CD [12], while another study reported lower 25(OH)D concentration in children with CD compared to those with UC, even though the mean 25(OH)D concentration was normal for the two IBD sub-types [11]. Two studies that defined vitamin D deficiency using a cut-off value of 37.4 nmol/L (15 ng/mL) reported prevalence rates of 16% for children with CD [10] and 10.8% for children and adolescents with IBD [8]. Three studies have investigated the vitamin D status of children and adolescents using a 25(OH)D cut-off value of 50 nmol/L (20 ng/mL), similar to the current study. The first of these studies which was conducted in Australia [22] reported a 19% prevalence of vitamin D deficiency while the other two studies from the same center in New England, USA, reported prevalence rates of 34.6% [8] and 14.3%[9]. A similar study in healthy adolescents in New England that used a cut-off value of 50 nmol/L to define vitamin D deficiency reported a prevalence of 42.0% [23]. An analysis of the prevalence of vitamin D deficiency in the subtypes of IBD detected no significant differences between UC and CD at 25(OH)D cut-off values of 38 nmol/L or 50 nmol/L [8]. Thus, the results of studies investigating the vitamin D status of children and adolescents with IBD are limited and discordant.

Though the studies that characterized the prevalence of vitamin D deficiency in IBD have provided important information in this field, they have not proven that vitamin D deficiency is a feature of IBD as they did not compare their vitamin D data to those of healthy children and adolescents in a case-controlled research design. This is expressed in a recent call for more studies comparing the vitamin D status of patients with IBD to those of healthy controls [9].

The lack of a demonstration of subnormal vitamin D status in IBD compared to controls may be explained by the fact that in addition to oral intake, this prohormone is synthesized in the skin through exposure to ultra-violet radiation. Hence, dietary intake of vitamin D is not necessarily required to maintain normal vitamin D status in individuals who maintain adequate exposure to sunlight.

There was neither a significant difference in serum 25(OH)D concentration between IBD subjects and controls, nor between IBD subjects with active vs. quiescent disease.

In contrast, IBD subjects with elevated ESR had significantly lower 25(OH)D concentration than IBD subjects with normal ESR values. When compared to controls, subjects with IBD and normal ESR level had similar 25(OH)D concentration as controls, whereas subjects with IBD and elevated ESR had significantly lower 25(OH)D than controls (65.3±28.0 nmol/L vs. 49.54±25.23, p = 0.045). This finding supports previous reports of an independent association between ESR and lower 25(OH)D concentration in IBD [8], [14]. However, to the best of our knowledge, this is the first study to report significantly lower 25(OH)D concentration in patients with IBD and elevated ESR compared to healthy controls.

### Adiposity and 25-hydroxyvitamin D

Obesity occurs in IBD despite the strong association between IBD and growth retardation [24]. A recent study of nearly 1600 children reported an obesity rate of 20% in children with IBD; and a rate for overweight status that is similar to that of the general population at nearly 30% [25]. The control group in this study had a non-significantly higher BMI z-score than the subjects with IBD. To determine the effect of adiposity on 25(OH)D concentration, we stratified the subjects and controls into normal-weight and overweight/obese groups based on BMI criteria(Figure 1). Even though there was no difference in mean serum 25(OH)D between the IBD patients and controls (Table 1), upon stratification into BMI sub-groups, the normal-weight controls had significantly higher 25(OH)D concentration compared to the overweight/obese IBD patients (p = 0.031). In contrast, serum 25(OH)D concentration was similar between the normal-weight IBD and the overweight/obese IBD patients (p = 0.883). The normal-weight controls had significantly higher 25(OH)D concentration compared to the normal-weight IBD subjects (p = 0.023), but there was neither a difference in serum 25(OH)D concentration between the overweight/obese controls vs. the overweight/obese IBD subjects, nor between normal-weight IBD vs. overweight/obese IBD.

We and others have shown that increased adiposity is associated with vitamin D deficiency [26], [27]. The mechanism of this association is unclear, however, proposed causative factors include poor nutrition, inadequate exposure to sunlight, and the sequestration of vitamin D in fat stores in overweight/obese individuals [28]. Interestingly, adiposity had no effect on the vitamin D status of our IBD cohort. More research is needed to determine the adiposity threshold necessary to induce significant reduction in serum 25(OH)D concentration [9] through processes such as the sequestration of vitamin D in fat stores in patients with IBD [28].

### Strengths and limitations

This study has some limitations. First, the cross-sectional study design limits causal inference on the effects of seasons, race, and adiposity on vitamin D status. Second, we did not administer a food-recall to accurately determine dietary vitamin D intake. Third, we did not exhaustively evaluate the components of the complex vitamin D metabolic pathway, such as parathyroid hormone (PTH), 1,25-dihydroxyvitamin D, 24,25-dihydroxyvitamin D, and vitamin D receptor activity. This is important because PTH could be elevated in states of vitamin D deficiency and hypocalcemia, while 24,25-dihyroxyvitamin D could be elevated in states on increased vitamin D degradation. However, 25(OH)D is the major circulation form of vitamin D and its stability in plasma, and a long half-life of >15 days makes it a highly sensitive and specific marker of vitamin D status [29]. Fourth, our control group was younger than the IBD cohort: such a difference could potentially influence our results, however, earlier studies found no relationship between age and 25(OH)D concentration in children and adolescents with IBD [8], [13]. Fifth, we did not adjust for the effect of pubertal maturation on 25(OH)D concentration, however, others have shown that pubertal maturation does not influence vitamin D concentration in IBD [30]. Finally, our results were derived from a single tertiary care center in the northern United States located at latitude 42°N. Therefore, we are uncertain that our results are generalizable to other centers, countries, and geographical latitudes.

The unique strength of this study is its case-controlled design which enabled us to evaluate a large cohort of patients with IBD and compare their results to a control group. This large sample size enabled us to detect subtle differences between the groups of interest. Our sample contained a fair representation of the fractional composition of each of the major racial groups in Central Massachusetts, thus enabling us to analyze the effects of differential insolation on racial groups. The control group was randomly selected using a structured randomization scheme. This study was conducted exclusively amongst subjects living in the same geographical latitude (42°N), thus ensuring uniformity of exposure to solar radiation. Phlebotomy was performed at different seasons of the year, thus ensuring that seasonality did not confound our results. Furthermore, the relationship between duration of IBD and 25(OH)D was analyzed, and effect of disease severity on 25(OH)D concentration in IBD was analyzed and compared to controls. All anthropometric data were expressed as z-score, and the analyses were adjusted for covariates.

## Conclusions

There was no difference in mean serum 25(OH)D concentration between children and adolescents with IBD and controls. IBD subjects with elevated ESR had significantly lower serum 25(OH)D concentration compared to the healthy controls, and subjects with IBD and normal ESR level. This finding of vitamin D deficiency in subjects with IBD and elevated ESR suggests that inflammation is a risk factor for vitamin D deficiency in IBD. Therefore, it may be prudent to closely monitor patients with IBD and elevated ESR for vitamin D deficiency.
